# Comparative Effectiveness of Second-Line Targeted Therapies for Metastatic Renal Cell Carcinoma: A Systematic Review and Meta-Analysis of Real-World Observational Studies

**DOI:** 10.1371/journal.pone.0114264

**Published:** 2014-12-10

**Authors:** Daniel Y. Heng, James Signorovitch, Elyse Swallow, Nanxin Li, Yichen Zhong, Paige Qin, Daisy Y. Zhuo, Xufang Wang, Jinhee Park, Sotirios Stergiopoulos, Christian Kollmannsberger

**Affiliations:** 1 Department of Medical Oncology, Tom Baker Cancer Center, Alberta Health Services Cancer Care, University of Calgary, Calgary, Canada; 2 Analysis Group, Inc., Boston, Massacusetts, United States of America; 3 Novartis Pharmaceuticals Corporation, East Hanover, New Jersey, United States of America; 4 Department of Oncology, British Columbia Cancer Agency, Vancouver, Canada; National Cancer Centre, Singapore

## Abstract

**Objective:**

The optimal sequencing of targeted therapies for metastatic renal cell carcinoma (mRCC) is unknown. Observational studies with a variety of designs have reported differing results. The objective of this study is to systematically summarize and interpret the published real-world evidence comparing sequential treatment for mRCC.

**Methods:**

A search was conducted in Medline and Embase (2009–2013), and conference proceedings from American Society of Clinical Oncology (ASCO), ASCO Genitourinary Cancers Symposium (ASCO-GU), and European Society for Medical Oncology (ESMO) (2011–2013). We systematically reviewed observational studies comparing second-line mRCC treatment with mammalian target of rapamycin inhibitors (mTORi) versus vascular endothelial growth factor (VEGF) tyrosine kinase inhibitors (TKI). Studies were evaluated for 1) use of a retrospective cohort design after initiation of second-line therapy, 2) adjustment for patient characteristics, and 3) use of data from multiple centers. Meta-analyses were conducted for comparisons of overall survival (OS) and progression-free survival (PFS).

**Results:**

Ten studies reported OS and exhibited significant heterogeneity in estimated second-line treatment effects (I^2^ = 68%; P = 0.001). Four of these were adjusted, multicenter, retrospective cohort studies, and these showed no evidence of heterogeneity (I^2^ = 0%; P = 0.61) and a significant association between second-line mTORi (>75% everolimus) and longer OS compared to VEGF TKI (>60% sorafenib) (HR = 0.82, 95% CI: 0.68 to 0.98) in a meta-analysis. Seven studies comparing PFS showed significant heterogeneity overall and among the adjusted, multicenter, retrospective cohort studies. Real-world observational data for axitinib outcomes was limited at the time of this study.

**Conclusions:**

Real-world studies employed different designs and reported heterogeneous results comparing the effectiveness of second-line mTORi and VEGF TKI in the treatment of mRCC. Within the subset of adjusted, multicenter observational studies, second-line use of mTORi was associated with significantly prolonged survival compared with second-line use of VEGF TKI.

## Introduction

Renal cell carcinoma (RCC) has a lifetime risk of approximately 1–2%, with one third to one half of cases presenting with or progressing to metastatic disease (mRCC) [1], [2]. The prognosis for mRCC is poor, with a historical 5-year survival rate of approximately 10% [3]. During the past decade, the advent of targeted therapies has significantly improved patient outcomes in mRCC. Seven targeted therapies are currently in use: the vascular endothelial growth factor (VEGF) tyrosine kinase inhibitors (TKIs) sorafenib, sunitinib, pazopanib, and axitinib, the VEGF-directed monoclonal antibody bevacizumab, and the mammalian target of rapamycin inhibitors (mTORis) everolimus and temsirolimus. Guidelines recommend treatment initiation with a VEGF TKI for most patients. However, the majority will eventually fail their first line treatment due to disease progression or intolerance. Sequential treatment with subsequent lines of VEGF TKI or mTORi is the current standard of care for mRCC [4]. However, there is no consensus on the optimal sequencing of targeted therapies after the failure of first-line VEGF TKI.

Evidence from available randomized clinical trials does not fully inform later-line treatment choices. The mTORi everolimus has shown superior PFS compared to placebo in the second-line setting, but has not been compared to other second-line targeted therapies in a completed randomized trial [5]. Sorafenib demonstrated comparable progression-free survival (PFS) and superior overall survival (OS) to temsirolimus [6] and inferior PFS compared with axitinib in the second-line setting [7]. However, no other randomized comparisons of targeted therapies are available in the second-line setting. In addition, randomized trials in mRCC have not directly demonstrated impacts on OS, due to crossovers between treatment arms following disease progression. Given the large number of treatment options for mRCC following the failure of a first targeted therapy, the comparative effectiveness of different sequential treatment strategies for mRCC, especially in terms of OS, is of high interest to physicians and patients.

To address this need for comparative evidence, a number of observational studies have been conducted to compare outcomes among different mRCC treatment sequences. The results of these studies have been mixed. Some have associated prolonged PFS or OS with second-line mTORi versus VEGF TKI [8], others with VEGF TKI versus mTORi [9]; others have found no significant differences among second-line treatments [10]. It is possible that differences across these studies could be due to heterogeneity in data sources, study designs and analytical methods. In addition, observational studies may be subject to varying levels of confounding and selection bias due to the lack of randomization [11].

When properly conducted and reported, observational studies can provide a valuable complement to clinical trial evidence in comparative effectiveness research by providing results applicable to broader, more inclusive populations that reflect real-world practice, and by comparing longer-term clinical outcomes such as OS. The differing results among currently available observational studies in mRCC present a challenge to decision makers who are interested in considering real-world evidence.

The present study systematically summarizes and interprets the published real-world evidence comparing OS and PFS for sequential treatment with VEGF TKI-mTORi versus VEGF TKI-VEGF TKI in mRCC patients. Since most patients receive a VEGF TKI in the first-line setting, and many studies do not adequately represent third-line treatment, we focused on comparisons of second-line treatment outcomes as a practical and meaningful first step in understanding the comparative effectiveness of treatment sequences. In addition, since most studies report only class-level treatment groups, we further focused on second-line mTORi versus second-line VEGF TKI at the class level. The objectives of this study are to assess whether the comparative evidence demonstrates significant heterogeneity across studies and to obtain consensus estimates of comparative effectiveness using meta-analysis when studies are suitably similar.

## Materials and Methods

### Systematic Literature Review

A systematic literature review was conducted using Medline and Embase (2009–2013), and conference proceedings from American Society of Clinical Oncology (ASCO), ASCO Genitourinary Cancers Symposium (ASCO-GU), and European Society for Medical Oncology (ESMO) (2011–2013). These date ranges are intended to capture publications of real-world data following the approval of mTORi in 2009, and to capture recent real-world data presented at conferences but not yet published in manuscript form. Search queries are included in the S1 Appendix in S1 File. We followed the Preferred Reporting Items for Systematic Reviews and Meta-Analysis (PRISMA) guidelines in designing, performing, and reporting the systematic review (S1 Checklist in S1 File) [12]. Included studies were required to: 1) be observational (i.e., non-randomized), 2) compare mTORi versus VEGF TKI as second-line treatments for mRCC, 3) report PFS or OS outcomes, and 4) be published in English. Reviews, case reports, economic models, analyses of randomized trials and other studies not reporting analyses of real-world data were excluded. When multiple analyses were identified using the same data source, only the analysis based on the most recent data was included. When peer-reviewed publications and conference presentations were identified for the same analysis, the conference presentation was excluded. The systematic literature review was conducted on September 3^rd^, 2013. Two researchers (YZ and PQ) independently applied the selection criteria, extracted the relevant data into a data collection spreadsheet with prepared fields, and assessed the quality of each included study; a third party (NL) was consulted to arbitrate disagreement.

### Assessment of Study Designs

In order to evaluate the reliability of comparative evidence, a pre-planned assessment of study designs was conducted. Included studies were classified according to criteria derived from the Newcastle-Ottawa Quality Assessment Scale for Cohort Studies [13].

1. Use of a retrospective cohort design after second-line treatment initiation. In a retrospective cohort design, inclusion criteria are applied only to patient history up to and including the exposure of interest, in this case the initiation of a second-line targeted therapy. All patients meeting the inclusion criteria are then followed, retrospectively, as long as possible for outcome events (progression or death) or censoring due to the end of follow-up. A common departure from retrospective cohort designs occurs when a patient’s inclusion in the study depends on events occurring after the exposure of interest, such as initiating a later-line treatment. This results in immortal time bias, which will bias comparative treatment effects to an unknown degree and direction [14]. The Newcastle-Ottawa scale does not explicitly include this criterion, however it is implicit in the classification of a study as a cohort study. In addition, study designs that are not valid retrospective cohorts would fail to satisfy the “representativeness of the exposed cohort” and the “adequacy of follow-up” items in the Newcastle-Ottawa scale. Therefore, the present review considered studies using a retrospective cohort design more reliable than those that did not.
2. Adjustment for patient characteristics. Non-randomized treatment groups, as are found in observational studies, may have different patient characteristics prior to starting treatment. Such differences can result in confounding bias, i.e. differences outcomes between treatment groups that are due to differences in patient characteristics, such as demographics, severity, prognostic factors, rather than to treatment effects. The risk of confounding bias may be reduced by adjustment for pre-treatment characteristics, such as in a multivariable regression model [15], [16]. In the present review, we assessed whether or not each study reported adjusted comparative analyses, and summarized the patient characteristics included in the adjustment. Comparative analyses that do not adjust for baseline differences would fail to satisfy the comparability of cohorts criterion in the Newcastle-Ottawa scale. Adjusted results were considered more reliable than unadjusted results in the present review.
3. Inclusion of data from multiple study centers. Multicenter studies are more likely to be representative and generalizable to broader populations, and are therefore considered more reliable than single-center studies [16], [17] In addition, treatment patterns at a single center may consistently channel particular patient profiles to particular treatments, which can result in confounding biases that are difficult to address via adjustment for patient characteristics. The Newcastle-Ottawa scale includes an assessment of the representativeness of the cohorts. In the present review, multicenter studies were considered more reliable than single-center studies.

Additional items from the Newcastle-Ottawa scale, including ascertainment of exposure, methods of outcome assessments and reporting of follow-up, were also evaluated.

### Meta-Analyses

Estimated treatment effects of second-line mTORi versus VEGF TKI were synthesized for OS and PFS across all identified studies using meta-analysis. Treatment effects were measured as hazard ratios (HRs). Pooled HRs and associated 95% confidence intervals (CIs) and P values were estimated under a random effects model. Separate meta-analyses were then applied to the subgroup of adjusted, multicenter, retrospective cohort studies (i.e., studies meeting all three criteria described above). When studies did not report HRs, they were imputed based on reported medians and associated 95% CIs for time to event and a constant hazard assumption. In each meta-analysis, heterogeneity was assessed using I^2^ and tested with Cochran's Q statistic and its associated P value. Small study bias was also assessed using funnel plots and Egger’s tests. Meta-analyses were conducted using the R software [18].

## Results

**Figure 1:**
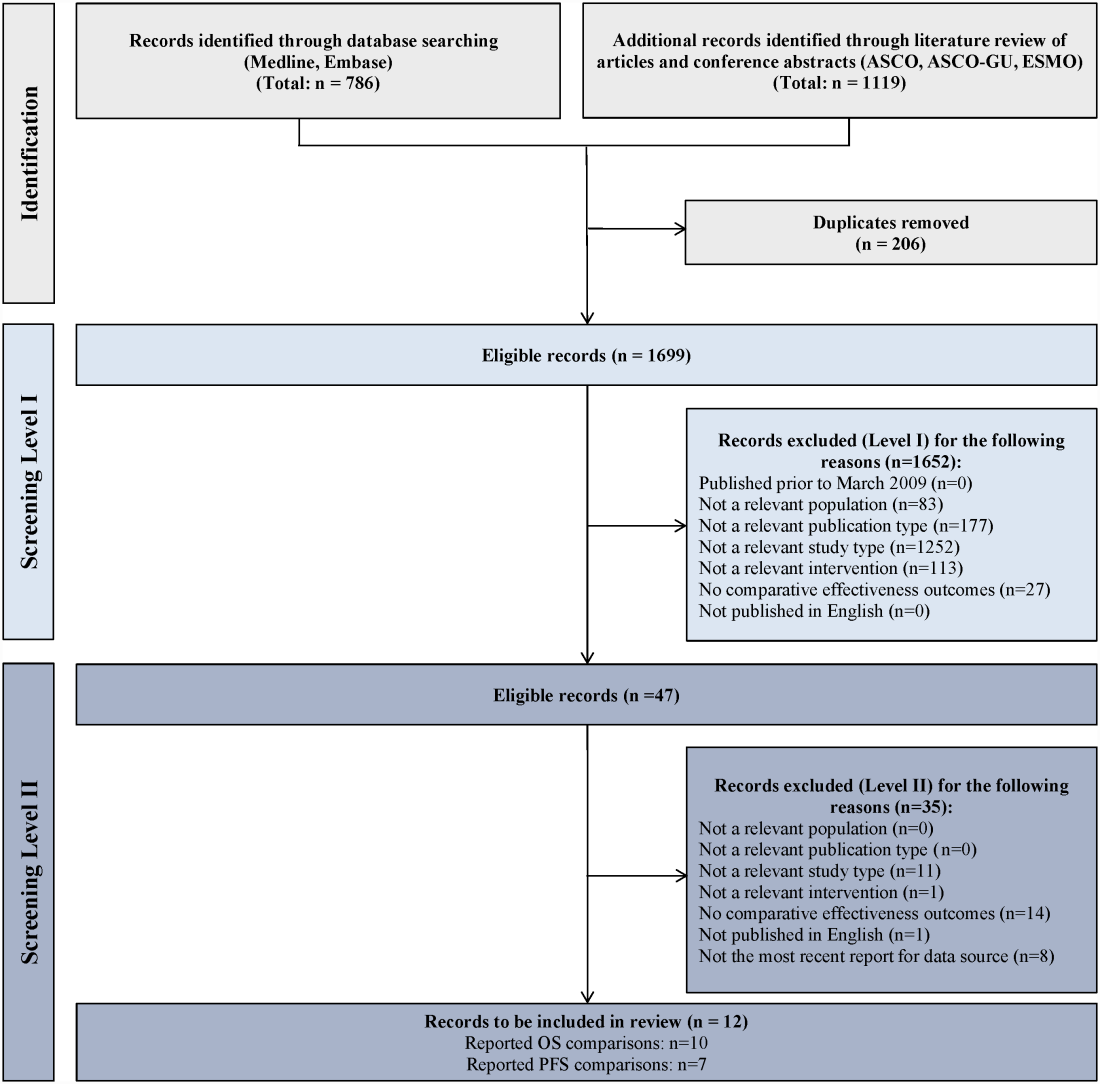
PRISMA diagram of selected records.

The systematic literature review identified 12 studies meeting all inclusion criteria: 6 peer-reviewed journal publications [8]–[10], [19]–[21] and 6 conference abstracts/posters [22]–[27] (Fig. 1). Among these studies, 10 reported treatment effects on OS and 7 reported effects on PFS and were subsequently included in further analyses for OS and PFS, respectively. Studies reporting OS included a pooled total of 2,228 patients: 961 patients who received second-line mTORi and 1,267 patients who received second-line VEGF TKI. Studies reporting PFS included a pooled total of 1,926 patients: 916 patients who received second-line mTORi and 1,010 patients who received second-line VEGF TKI.

### Studies reporting OS

Study designs differed substantially among the 10 studies reporting OS (Table 1). Seven employed a retrospective cohort design [8], [10], [20]–[24]. The 3 studies that departed from a retrospective cohort design did so by requiring patients to receive a third-line therapy after the initiation of second-line treatment [9], [19], [27], resulting in immortal time bias for the effects of second-line treatment. Seven out of the 10 studies reported adjusted treatment effects [8]–[10], [19]–[22]. Patient characteristics used for multivariable adjustment are listed in Table 2. With the exception of one claims-based study [10], the studies adjusted for similar mRCC prognostic factors, including the Memorial Sloan-Kettering Cancer Center (MSKCC) score [28], the Heng et al. criteria [29] or their components (MSKCC score components: Karnofsky performance status (KPS), time from diagnosis to therapy, serum lactate dehydrogenase level, hemoglobin level, and corrected serum calcium; Heng et al. criteria components: KPS, time from diagnosis to therapy, hemoglobin level, corrected serum calcium, neutrophil level, and platelet level). Eight out of the 10 studies were conducted in multiple centers in North America and Europe [8]–[10], [19], [20], [22], [23], [27]; the 2 identified single-centered studies were conducted in South Korea [21] and Spain [24]. Four studies met all 3 criteria (i.e., were multicenter, adjusted, retrospective cohort studies) and were considered for separate meta-analyses [8], [10], [20], [22]. The 10 studies differed in the allowed reasons for discontinuing first-line therapy, ranging from requiring progression on first-line [20] to broader definitions of first-line treatment failure including progression, non-response and lack of tolerability [8], [19], [21] (Table 1). Additional criteria included in the Newcastle-Ottawa scale either did not differentiate among studies or were not relevant for this review of OS and PFS. In particular, in all studies patients were necessarily free of the outcomes (observed progression or death) at the start of second-line therapy. None of the studies included outcome assessments that were blinded to treatment group. No studies provided a detailed accounting of all subjects lost to follow-up, however all studies used statistical methods appropriate for random censoring. In all studies, ascertainment of exposure was based on secure records (medical records or claims).

**Table 1:** Studies comparing OS and PFS with VEGF TKI-mTORi versus VEGF TKI-VEGF TKI (HR<1 favors second-line mTORi versus VEGF TKI).

Study	DataSource	InclusionCriteria	mTORiincluded	VEGFTKIincluded	RetrospectiveCohort	Adjustment	Multicenter	N,mTORi	N,VEGFTKI	OS HR(95% CI)^a^	PFS HR(95% CI)^b^
Buschet al.2011	Medicalrecords from2 centers inGermany	Progression on first-line VEGF TKI	Everolimus	Sunitinibandsorafenib	Y	Y^d^	Y	62	46	0.79(0.43, 1.45)	0.86(0.57–1.28)^d^
Chenet al.2012	US claimsdata	Received sunitinib	Everolimus	Sorafenib	Y	Y	Y	117	65	1.03(0.59, 1.79)	N/A
Henget al.2012^c^	Internationalregistry(Canada,UnitedStates,Singapore, andDenmark)^b^	Received first-line VEGF TKI	Everolimusandtemsirolimus	Sunitinibandsorafenib	Y	Y^d^	Y	277	541	0.84(0.67, 1.06)	1.18(0.92–1.5)^d^
Wonget al.2013	Nationwidechart reviewin the UnitedStates	Failed first-line VEGF TKI	Everolimus	Sorafenib	Y	Y	Y	233	123	0.65(0.42, 0.99)	0.75(0.53–1.07)
Parket al.2012	Medicalrecords froma singlecenter inSouth Korea	Failed first-line VEGF TKI	Everolimusandtemsirolimus	Sunitinibandsorafenib	Y	Y^d^	N	42	41	1.71(0.86, 3.4)	1.03(0.62–1.69)^d^
Goreet al.2013	Multicenter,Australia,Brazil,Canada,Europe,United States	Received first-line sunitinib in a randomized trial	Everolimus,temsirolimus,SirolimusandSGN-75	Sunitinibandsorafenib	Y	N	Y	42	171	1.05(0.71, 1.54)	N/A
Harrisonet al.2012	Multicenter,United States	Patients alive since January 2007 and diagnosed between January 1, 2007, and February 7, 2011	Notspecified	Notspecified	N	N	Y	33	32	3.13 (0.96, 10.22)	N/A
Ruizet al.2013	Single-institution,Spain	Received at least 1 line of target therapy between 2007 and 2011	Everolimusandtemsirolimus	Sunitinib,sorafenib,bevacizumab,pazopanib,axitinib^e^,dovitinib	Y	N	N	19	34	1.10(0.56, 2.17)	N/A
Buschet al.2013	Medicalrecords from2 centers inGermany	Failure of first-line VEGF TKI	Everolimusandtemsirolimus	Sunitinibandsorafenib	N	Y^d^	Y	41	62	0.86(0.51, 1.44)	0.76(0.43–1.35)^d^
Iacovelliet al.2013	Medicalrecords frommultiplecenters inItaly	Patients consecutively treated with 3 targeted therapies	Everolimusandtemsirolimus	Sunitinibandsorafenib	N	Y	Y	95	152	2.59(1.59, 4.22)	N/A
Elaidiet al.2013	Medicalrecords from7 centers inEurope	Received VEGF TKI-VEGF TKI or VEGF TKI-mTORi	Everolimusandtemsirolimus	Sunitinib,sorafenib,pazopanib,axitinib^e^	Y	Y	Y	123	118	N/A	1.56(1.11–2.22)
Signorovitchet al.2013	Chart review,multicenter,United States	Started second-line targeted therapy in 2010 or later	Everolimusandtemsirolimus	Sunitinib,sorafenib,pazopanib,axitinib^e^	Y	Y	Y	138	79	N/A	0.74(0.48, 1.15)

CI, confidence interval; HR, hazard ratio; mTORi, mammalian target of rapamycin inhibitor; N/A, not available; OS, overall survival; PFS, progression-free survival; VEGF TKI, vascular endothelial growth factor tyrosine kinase inhibitor.

a OS HRs and 95% CIs were imputed for Harrison et al. 2012, Gore et al. 2013, and Ruiz et al. 2013.

b PFS HRs and 95% CIs were imputed for Heng et al. 2012 and Busch et al. 2013.

c Heng et al. 2012 used prospective data collection. However, the comparative analysis was performed retrospectively.

d The PFS results for Busch et al. 2011, Heng et al. 2012, Park et al. 2012, and Busch et al. 2013 were unadjusted. Y in column adjustment refers to the OS results.

e Only one patient received axitinib in Elaidi et al. 2013. The number of patients who received axitinib was not reported in Ruiz et al. 2013. The number receiving axitinib was n = 10 in Signorovitch et al. 2013 (personal communication).

**Table 2:** Patient characteristics used for multivariable adjustment.

Study	Patient characteristics
**Busch et al. 2011**	MSKCC risk group, prior immunotherapy, first-line sunitinib, primary first-line VEGF TKI resistance, second-line primary resistance and more
**Chen et al. 2012**	Sex, age, payer type, region, treating physician’s academic affiliation and specialty, site of metastases, second-line medication possession ratio, and comorbidity at the time of second-line treatment
**Heng et al. 2012**	Heng et al. criteria, non-clear cell histology, and nephrectomy status
**Wong et al. 2013**	Age, gender, race, whether metastasis was present at initial diagnosis, duration of mRCC, type of first targeted therapy, response to and duration of first targeted therapy, treatments received before first targeted therapy, comorbidities, number and sites of metastasis, sarcomatoid differentiation, non-clear-cell RCC, and KPS, as well as physician’s practice setting and year of practice
**Park et al. 2012**	Age, absolute neutrophil count, platelet count, Karnofsky performance status, time from diagnosis to treatment, corrected calcium level, first-line VEGF TKI
**Busch et al.2013**	MSKCC risk group, prior immunotherapy, first-line sunitinib, primary first-line VEGF TKI resistance, toxicity of second-line
**Iacovelli et al. 2013**	Initial prognostic group by MSKCC, and primary resistance at first-line

**Figure 2:**
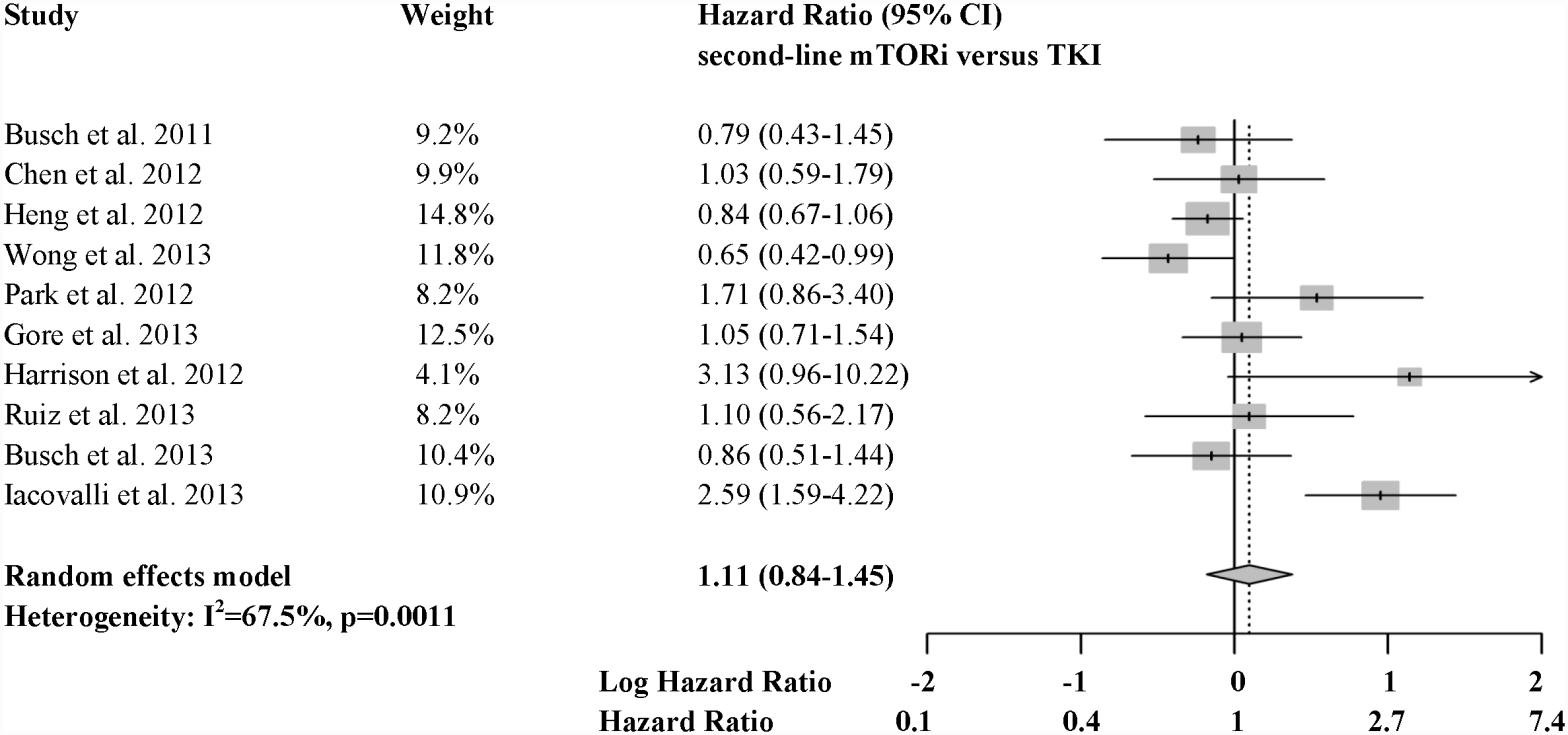
Forest plots of 10 studies reporting comparative OS results.

**Figure 3:**
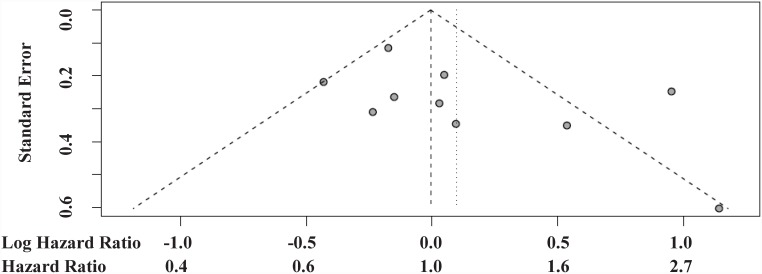
Funnel plots of 10 studies reporting comparative OS results.

Hazard ratios for death comparing second-line mTORi versus VEGF TKI ranged from 0.65 to 3.13 across the 10 identified studies. A meta-analysis pooling all of these HRs exhibited significant heterogeneity, with over twice as much variability arising from between study differences as from within studies (I^2^ = 68%; P = 0.001; Fig. 2). No evidence of small study reporting bias was detected by the funnel plot (Fig. 3) or the Egger’s test (P = 0.146). No significant difference in OS was identified between treatment sequences in this overall meta-analysis (HR = 1.11, 95% CI 0.84–1.45, P = 0.491), and, more importantly, the pooled effect estimate is difficult to interpret due to the significant heterogeneity.

**Figure 4:**
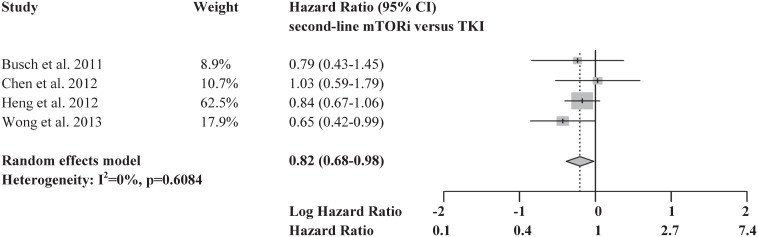
Forest plots of 4 studies meeting all reliability criteria reporting comparative OS results.

**Figure 5:**
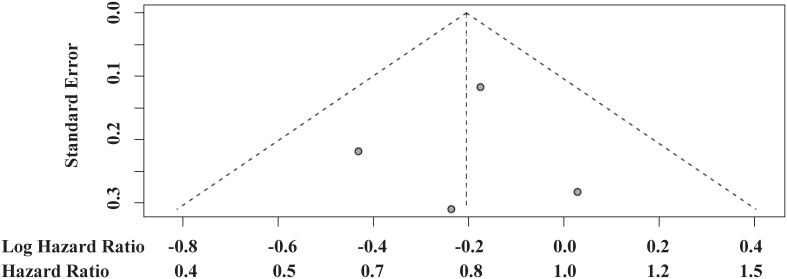
Funnel plots of 4 studies meeting all reliability criteria reporting comparative OS results.

A meta-analysis including only the 4 adjusted, multicenter, retrospective cohort studies [8], [10], [20], [22] was also performed (Fig. 4). These 4 studies included a total of 1,464 patients, constituting over half of the total number of patients in all 10 studies. 689 of these patients received mTORi (>75% everolimus) and 775 patients received VEGF TKI therapy (>60% sorafenib, no axitinib) in the second-line. There was no evidence of heterogeneity in the comparative effects estimates among these 4 studies (I^2^ = 0%; P = 0.608). The funnel plot was symmetrical, indicating no evidence of publication bias (Fig. 5; Egger’s test was not performed due to the small number of studies). In a meta-analysis of these four studies meeting reliability criteria, second-line mTORi was associated with significantly prolonged OS compared with VEGF TKI, corresponding to an 18% reduction in the hazard of death (HR = 0.82, 95% CI 0.68 to 0.98, P = 0.028).

As a sensitivity analysis, we further investigated the impact of one additional study, Park et al. [21] which used an adjusted retrospective cohort design, but was conducted in a single-center in South Korea (N = 42 patients with mTORi and N = 41 patients with VEGF TKI as second-line treatment). This study reported numerically shorter OS for second-line mTORi compared to VEGF TKI (adjusted HR = 1.71, 95% CI 0.86 to 3.40, P = 0.125), which, despite the wide confidence interval and small sample size, was significantly different from the pooled HR among the 4 adjusted, multicenter, retrospective cohort studies (P = 0.004). When Park et al. was pooled with these 4 studies, the resulting HR for mTORi versus VEGF TKI increased from 0.82 to 0.85 and the confidence interval increased in width (HR = 0.85, 95% CI 0.72 to 1.02, P = 0.082).

### Studies reporting PFS

The 7 studies reporting PFS differed substantially in their designs: 6 used a retrospective cohort design [8], [20]–[22], [25], [26], 4 used multivariable adjustment [8], [21], [25], [26] and 6 were multicenter studies [8], [19], [20], [22], [25], [26]; 3 met all three criteria [8], [25], [26]. A meta-analysis pooling all 7 studies identified significant heterogeneity (I^2^ = 57%; P = 0.031), and did not find a significant difference in PFS between second-line mTORi and second-line VEGF TKI (HR = 0.98, 95% CI 0.78 to 1.22, P = 0.827). Significant heterogeneity remained when the analysis was limited to the 3 adjusted, multicenter, retrospective cohort studies (I^2^ = 81%; P = 0.005) [8], [25], [26], and the pooled analysis of these studies did not identify any treatment differences (HR = 0.96, 95% CI 0.58 to 1.59, P = 0.876). Among them, Signorovitch et al, 2013 and Wong et al, 2013 reported numerically longer PFS for second-line mTORi compared to VEGF TKI (HR = 0.74, 95% CI 0.48 to 1.15; HR = 0.75, 95% CI 0.53 to 1.07); Elaidi et al, 2013 reported significantly shorter PFS for second-line mTORi compared to VEGF TKI (HR = 1.56, 95% CI 1.11 to 2.22).

## Discussion

This study systematically reviewed and synthesized real-world comparative evidence for second-line mTORi versus VEGF TKI in the treatment of mRCC. Study designs and patient populations varied across studies, and this variation was reflected in significant heterogeneity in the estimated comparative effects on OS and PFS. Pooling all of the studies together, there was no evidence of a difference between mTORi and VEGF TKI in the second-line setting. However, more importantly, the high level of heterogeneity indicated that it was not possible to draw a single comparative conclusion from the diverse collection of all identified studies.

When synthesizing comparative evidence, it is important to consider the reliability of the included studies in addition to their heterogeneity. To this end, we applied three criteria, adapted from the Newcastle-Ottawa scale, to identify studies with the most reliable comparative study designs [13]. First, we required studies to follow a retrospective cohort design that imposed inclusion criteria only up to the initiation of second-line therapy, and then followed all included patients as long as possible for outcomes. This is similar to requiring an “intent-to-treat” approach in clinical trials. The three studies that did not meet this criterion required patients to initiate third-line therapy [9], [19], [27], and therefore excluded large proportions of second-line patients who did not reach third line during the study period due to loss to follow-up, continuation of second-line treatment at the time of chart review, death during second-line therapy, or other reasons. These patients contain valuable information about second-line treatment outcomes. While designs that exclude these patients, by requiring three lines of treatment, provide valuable retrospective descriptions of patient treatment sequences, they are not valid designs for comparing the effectiveness of second-line treatment choices [14]. Indeed, such study populations cannot be identified in clinical practice at the time second-line treatment decisions are made because future use of third-line treatment is unknown at that point. As a second criterion, we required studies to report comparative outcomes that were adjusted for patients’ characteristics prior to the initiation of second-line treatment. Comparative studies that do not adjust for baseline characteristics could be biased by avoidable baseline differences [15], [16]. Finally, we required studies to draw data from multiple treatment centers, as such studies are considered more representative and generalizable than single-center studies [16], [17]. Studies not meeting these three criteria may provide valuable descriptive evidence, and could meet general quality criteria for reporting of observational studies, but do not provide the same level of comparative evidence as studies that do meet the criteria. On a per-patient basis, the majority of the evidence identified in our systematic review met all three of these reliability criteria. Additional items from the Newcastle-Ottawa scale were also evaluated, but did not differentiate among studies.

It is notable that after focusing the meta-analysis on adjusted, multicenter, retrospective cohort studies, there was no evidence of heterogeneity in estimated second-line treatment effects on OS. This suggests that these four studies, although based on diverse data sources including a prospective multi-national registry, medical records from Germany, a retrospective chart review in the US and US claims data, are estimating the same underlying association between second-line treatment and OS. The pooled estimate from these studies showed a significant association between use of mTORi and prolonged OS compared with VEGF TKI in the second-line setting. The magnitude of the difference was clinically significant, representing an 18% decrease in the hazard of death associated with second-line mTORi.

One additional study that employed an adjusted, retrospective cohort design, but was conducted at a single center in South Korea, was considered in a sensitivity analysis. Despite including fewer than 100 patients, this study showed a significantly different and opposite association between second-line treatment and OS than the pooled analysis of the four studies meeting all three criteria. It was not possible to assess whether this difference was due to factors affecting the single center in South Korea, or other potential differences. Nevertheless, inclusion of this study in the meta-analysis, along with the adjusted, multicenter, retrospective cohort studies, did not significantly change the hazard ratio for second-line mTORi versus VEGF TKI.

As observed for the comparative studies of OS, the full group of studies comparing PFS showed significant heterogeneity and no significant differences between second-line mTORi and VEGF TKI. However, even after focusing the meta-analysis of PFS on adjusted, multicenter, retrospective cohort studies, significant heterogeneity remained among the PFS comparisons. Potential reasons for greater heterogeneity in PFS were not clear. Results were consistent between two separate US-based chart reviews, which suggested longer PFS with second-line mTORi versus VEGF TKI [8], [26]. However, a multinational European study reported the opposite association [25]. It was not possible to reach a consensus conclusion about comparative effects on PFS by pooling these studies.

This review and meta-analysis of observational studies carries important limitations. The foremost limitation is that the meta-analyses are based on non-randomized treatment comparisons. The comparisons between drug classes may be confounded by differences in the types of patients treated with each class. Potential confounding factors may include, for example, differences in age, metastatic burden, RCC histology, performance status, response to first VEGF TKI, lab values (e.g., neutrophil count, platelet count, corrected calcium level) or composite risk scores (e.g., MSKCC or Heng et al. criteria). Study design features that depart from a retrospective cohort design, such as requiring the initiation of a 3rd-line treatment, could also introduce bias. Since the present study relied on published data, it was not possible to adjust for pre-specified characteristics at the patient level. We aimed to limit the potential for confounding in our meta-analyses by conducting sub-analyses of published studies that included more reliable comparative designs. However, even the included studies with the more reliable comparative designs and adjustment for important prognostic factors, may be confounded by unobserved differences in patient populations.

Only a well-conducted randomized trial can avoid the potential for confounding. However, little evidence from randomized controlled trials comparing mTORi to VEGF TKI in the second-line setting is currently available. A recent randomized controlled trial reported comparable PFS but significantly better OS for second-line use of sorafenib, a VEGF TKI, versus temsirolimus, an mTORi [6]. However, this study did not report subsequent treatments that were off-protocol, which might have influenced the results. Additionally, this study did not include everolimus, the mTORi used by the majority of the patients in the present study, or other VEGF TKIs (e.g., sunitinib); therefore, a comparative conclusion at the class level cannot be made. There are also potential limitations due to missing or inaccurate data obtained from real-world practice. In particular, assessments of progression may vary across practices and patients depending on visit schedules and the use and interpretation of imaging. The present study also pooled treatments at the class level, comparing mTORi vs. VEGF TKI, since most underlying studies did not report drug-specific results. However, there is evidence that individual drug effects can vary within these classes [7], [8], [10]. Future real-world research, with adequate sample sizes, will be valuable for understanding drug specific effects. In the present study, the majority of second-line mTOR use was everolimus and the majority of second-line VEGF TKI use was sorafenib.

This study also carries limitations inherent in meta-analysis. Though we conducted a systematic review of both peer-reviewed publications and conference proceedings, there is a possibility of publication bias, such as the selective reporting of significant findings. However, there is reason to believe that publication bias was absent or negligible in the present review. Following the advent of new targeted therapies for mRCC, there has been high interest in any real-world data on treatment outcomes with sequential targeted therapy. Indeed, most identified studies did not individually show statistically significant treatment differences. It should also be noted that this review included conference proceedings in addition to peer-reviewed publications. Conference proceedings may be subject to revision during peer review. On the other hand, conference proceedings often report more recent real-world data, which is important when studying the outcomes that reflect recently approved treatments, including everolimus in the second line setting and pazopanib in the first-line setting. At the time of this study, adequate real-world non-clinical trial evidence was not available for study of axitinib outcomes in the second-line setting and hence no axitinib-specific data were included. Whether axitinib would change the reported results remains to be seen in future studies. Finally, the present study did not compare outcomes among different 3^rd^-line treatment choices. An appropriate retrospective cohort design for comparing 3^rd^-line treatment outcomes would follow patients after initiation of 3^rd^-line treatment, and would adjust for patient characteristics available at the time of 3rd-line treatment initiation, including treatments received in the first and second line. Future real-world studies of 3^rd^-line treatment outcomes will be valuable.

## Conclusions

In this systematic review, real-world studies employed different designs and reported heterogeneous results comparing the effectiveness of second-line mTORi and VEGF TKI in the treatment of mRCC. Due to the high heterogeneity, it was not possible to draw a comparative conclusion from the full set of identified studies. In a sub-analysis of studies with more reliable designs for comparative analysis (i.e., adjusted, multicenter, retrospective cohort studies), second-line use of mTORi was associated with significantly prolonged OS compared with second-line use of VEGF TKI in the treatment of mRCC. Real-world outcomes for axitinib were not available at the time of this analysis, and should be considered in future studies. The present review demonstrates that study design should be considered when interpreting observational studies comparing treatment sequences in mRCC.

## Supporting Information

S1 File **S1 Appendix**, Full search strategy. **S1 Checklist**, PRISMA checklist.
(DOCX)

## References

[1] Gupta K, Miller JD, Li JZ, Russell MW, Charbonneau C (2008) Epidemiologic and socioeconomic burden of metastatic renal cell carcinoma (mRCC): a literature review. Cancer Treat Rev 34:193–205. 18313224 10.1016/j.ctrv.2007.12.001

[2] Cancer Research UK (2013) Kidney cancer incidence statistics.

[3] Lam JS, Leppert JT, Belldegrun AS, Figlin RA (2005) Novel approaches in the therapy of metastatic renal cell carcinoma. World J Urol 23:202–212. 15812574 10.1007/s00345-004-0466-0

[4] NCCN Guidelines (2014) NCCN Clinical Practice Guidelines in Oncology, Kidney Cancer (version 2.2014).

[5] Motzer RJ, Escudier B, Oudard S, Hutson TE, Porta C, et al (2008) Efficacy of everolimus in advanced renal cell carcinoma: a double-blind, randomised, placebo-controlled phase III trial. Lancet 372:449–456. 18653228 10.1016/S0140-6736(08)61039-9

[6] Hutson TE, Escudier B, Esteban E, Bjarnason GA, Lim HY, et al (2014) Randomized Phase III Trial of Temsirolimus Versus Sorafenib As Second-Line Therapy After Sunitinib in Patients With Metastatic Renal Cell Carcinoma. J Clin Oncol 32:760–767. 24297950 10.1200/JCO.2013.50.3961 PMC5569683

[7] Rini BI, Escudier B, Tomczak P, Kaprin A, Szczylik C, et al (2011) Comparative effectiveness of axitinib versus sorafenib in advanced renal cell carcinoma (AXIS): a randomised phase 3 trial. Lancet 378:1931–1939. 22056247 10.1016/S0140-6736(11)61613-9

[8] Wong MK, Yang H, Signorovitch JE, Wang X, Liu Z, et al. (2013) Comparative Outcomes of Everolimus, Temsirolimus and Sorafenib as Second Targeted Therapies for Metastatic Renal Cell Carcinoma: A U.S. Medical Record Review. Curr Med Res Opin. 10.1185/03007995.2013.871243 24329572

[9] Iacovelli R, Carteni G, Sternberg CN, Milella M, Santoni M, et al. (2013) Clinical outcomes in patients receiving three lines of targeted therapy for metastatic renal cell carcinoma: Results from a large patient cohort. Eur J Cancer. 10.1016/j.ejca.2013.02.032 23518211

[10] Chen CC, Hess GP, Liu Z, Gesme DH, Agarwala SS, et al (2012) Second-line treatment outcomes after first-line sunitinib therapy in metastatic renal cell carcinoma. Clin Genitourin Cancer 10:256–261. 22682982 10.1016/j.clgc.2012.04.006

[11] Egger M, Schneider M, Davey Smith G (1998) Spurious precision? Meta-analysis of observational studies. BMJ 316:140–144. 9462324 10.1136/bmj.316.7125.140 PMC2665367

[12] Liberati A, Altman DG, Tetzlaff J, Mulrow C, Gotzsche PC, et al (2009) The PRISMA statement for reporting systematic reviews and meta-analyses of studies that evaluate healthcare interventions: explanation and elaboration. BMJ 339:b2700. 19622552 10.1136/bmj.b2700 PMC2714672

[13] Wells GA, Shea B, O'Connell D, Peterson J, Welch W, et al. (2011) The Newcastle-Ottawa Scale (NOS) for assessing the quality of nonrandomized studies in meta-analysis.

[14] Lash TL, Cole SR (2009) Immortal person-time in studies of cancer outcomes. J Clin Oncol 27:e55–56. 19597013 10.1200/JCO.2009.24.1877

[15] Koepsell TD, Weiss NS (2003) Epidemiologic Methods: Studying the Occurrence of Illness: Oxford University Press.

[16] Elwood M (2007) Critical Appraisal of Epidemiological Studies and Clinical Trials: Oxford University Press.

[17] Meinert CL (2012) Clinical Trials: Design, Conduct and Analysis: Oxford University Press.

[18] R Core Team (2012) R: A language and environment for statistical computing. Vienna, Austria: R Foundation for Statistical Computing.

[19] Busch J, Seidel C, Erber B, Issever AS, Hinz S, et al (2013) Retrospective comparison of triple-sequence therapies in metastatic renal cell carcinoma. Eur Urol 64:62–70. 22999519 10.1016/j.eururo.2012.09.004

[20] Busch J, Seidel C, Kempkensteffen C, Johannsen M, Wolff I, et al (2011) Sequence therapy in patients with metastatic renal cell carcinoma: comparison of common targeted treatment options following failure of receptor tyrosine kinase inhibitors. Eur Urol 60:1163–1170. 21802830 10.1016/j.eururo.2011.07.037

[21] Park K, Lee JL, Park I, Park S, Ahn Y, et al (2012) Comparative efficacy of vascular endothelial growth factor (VEGF) tyrosine kinase inhibitor (TKI) and mammalian target of rapamycin (mTOR) inhibitor as second-line therapy in patients with metastatic renal cell carcinoma after the failure of first-line VEGF TKI. Med Oncol 29:3291–3297. 22460837 10.1007/s12032-012-0227-7

[22] Heng DYC, Lee J-L, Harshman LC, Bjarnason GA, Razak AR, et al (2012) A population-based overview of sequences of targeted therapy in metastatic renal cell carcinoma (mRCC). ASCO Meeting Abstracts 30:387.

[23] Gore ME, Hutson TE, Lin X, Korytowsky B, Lechuga MJ, et al. (2013) Treatment sequencing following first-line sunitinib in patients (pts) with advanced renal cell carcinoma (RCC). ESMO Meeting Abstract.

[24] Ruiz AL, Bolos MV, Viqueira A, Esteban E (2013) Outcome of metastatic renal cell carcinoma (mRCC) patients in the era of new targeted therapies. ESMO Meeting Abstract.

[25] Elaidi RT, Harbaoui AH, Beuselinck B, Eymard JC, Bamias A, et al. (2013) What is the best treatment option for second-line in long-responders to the first line TKI in metastatic renal cell carcinoma (mRCC) patients (pts): TKI-TKI or TKI-mTORi? Final results of a European retrospective study. ESMO Meeting Abstract.

[26] Signorovitch JE, Vogelzang NJ, Pal SK, Lin PL, George DJ, et al (2013) Comparative effectiveness of second-line targeted therapies for metastatic renal cell carcinoma: Analysis of two practice-based chart reviews. ASCO Meeting Abstracts 31:e15504.

[27] Harrison MR, George DJ, Walker MS, Hudson LL, Chen C, et al (2012) Outcomes of “real world” treatment for metastatic renal cell carcinoma (mRCC). ASCO Meeting Abstracts 30:406.

[28] Motzer RJ, Bacik J, Murphy BA, Russo P, Mazumdar M (2002) Interferon-alfa as a comparative treatment for clinical trials of new therapies against advanced renal cell carcinoma. J Clin Oncol 20:289–296. 11773181 10.1200/JCO.2002.20.1.289

[29] Heng DY, Xie W, Regan MM, Warren MA, Golshayan AR, et al (2009) Prognostic factors for overall survival in patients with metastatic renal cell carcinoma treated with vascular endothelial growth factor-targeted agents: results from a large, multicenter study. J Clin Oncol 27:5794–5799. 19826129 10.1200/JCO.2008.21.4809

