# Supplementary material for: Comparative Effectiveness of Second-Line Targeted Therapies for Metastatic Renal Cell Carcinoma: A Systematic Review and Meta-Analysis of Real-World Observational Studies
Source: PLoS One. 2014 Dec 10;9(12):e114264. doi: 10.1371/journal.pone.0114264 (PMC4262396; doi:10.1371/journal.pone.0114264)
Supplement: S1 File — S1 Appendix, Full search strategy. S1 Checklist, PRISMA checklist. (DOCX) [file pone.0114264.s001.docx]

**Appendix S1 – Full search strategy**

To identify real-world observational studies comparing OS and PFS between second-line mTORi and VEGF TKI in mRCC, the search terms used for Medline and Embase were:

("RCC" or "renal cell carcinoma" or "mRCC" or "metastatic renal cell carcinoma" or “advanced renal cell carcinoma” or “kidney cancer” or “renal cell cancer”) and ("targeted therapy" or "VEGF" or “vascular endothelial growth factor” or “TKI” or “tyrosine kinase inhibitor” or “bevacizumab” or “sorafenib” or “sunitinib” or “pazopanib” or “axitinib”) and (“targeted therapy” or “mTOR” or “mammalian target of rapamycin” or “everolimus” or “temsirolimus”) and (“sequence” or “sequential” or “line” or “lines” or “refractory”) and ("compare" or “compared” or “comparative” or “comparison” or “comparable” or “versus” or “vs” or “vs.” or “different” or “difference” or “same” or “similar” or “associate” or “relate” or “relationship” or “lead to” or “effect” or “hazard ratio” or “HR”). Search term used for ASCO, ASCO-GU, and ESMO were “RCC” or “Renal cell carcinoma”.

| **Checklist S1 PRISMA checklist.** | | | |
| --- | --- | --- | --- |
| **Section/topic** | **#** | **Checklist item** | **Reported on page #** |
| **TITLE** | | |  |
| Title | 1 | Identify the report as a systematic review, meta-analysis, or both. | Title (p.1) |
| **ABSTRACT** | | |  |
| Structured summary | 2 | Provide a structured summary including, as applicable: background; objectives; data sources; study eligibility criteria, participants, and interventions; study appraisal and synthesis methods; results; limitations; conclusions and implications of key findings; systematic review registration number. | Abstract (p. 2) |
| **INTRODUCTION** | | |  |
| Rationale | 3 | Describe the rationale for the review in the context of what is already known. | Introduction (p. 4-5) |
| Objectives | 4 | Provide an explicit statement of questions being addressed with reference to participants, interventions, comparisons, outcomes, and study design (PICOS). | Introduction (p. 6) |
| **METHODS** | | |  |
| Protocol and registration | 5 | Indicate if a review protocol exists, if and where it can be accessed (e.g., Web address), and, if available, provide registration information including registration number. | Methods: Systematic literature review (p. 6) |
| Eligibility criteria | 6 | Specify study characteristics (e.g., PICOS, length of follow-up) and report characteristics (e.g., years considered, language, publication status) used as criteria for eligibility, giving rationale. | Methods: Systematic literature review (p. 6-7) |
| Information sources | 7 | Describe all information sources (e.g., databases with dates of coverage, contact with study authors to identify additional studies) in the search and date last searched. | Methods: Systematic literature review (p. 6-7) |
| Search | 8 | Present full electronic search strategy for at least one database, including any limits used, such that it could be repeated. | Appendix S1 (File S1) |
| Study selection | 9 | State the process for selecting studies (i.e., screening, eligibility, included in systematic review, and, if applicable, included in the meta-analysis). | Methods: Systematic literature review, Meta-analyses (p. 6-7, p.9) |
| Data collection process | 10 | Describe method of data extraction from reports (e.g., piloted forms, independently, in duplicate) and any processes for obtaining and confirming data from investigators. | Methods: Systematic literature review (p. 6-7) |
| Data items | 11 | List and define all variables for which data were sought (e.g., PICOS, funding sources) and any assumptions and simplifications made. | Methods: Systematic literature review (p. 6-7) |
| Risk of bias in individual studies | 12 | Describe methods used for assessing risk of bias of individual studies (including specification of whether this was done at the study or outcome level), and how this information is to be used in any data synthesis. | Methods: Assessment of study design (p. 7-9) |
| Summary measures | 13 | State the principal summary measures (e.g., risk ratio, difference in means). | Methods: Meta-analyses (p. 9) |
| Synthesis of results | 14 | Describe the methods of handling data and combining results of studies, if done, including measures of consistency (e.g., I^2^) for each meta-analysis. | Methods: Meta-analyses (p. 9) |

Page 1 of 2

| **Section/topic** | **#** | **Checklist item** | **Reported on page #** |
| --- | --- | --- | --- |
| Risk of bias across studies | 15 | Specify any assessment of risk of bias that may affect the cumulative evidence (e.g., publication bias, selective reporting within studies). | Methods: Assessment of study design (p. 7-9) |
| Additional analyses | 16 | Describe methods of additional analyses (e.g., sensitivity or subgroup analyses, meta-regression), if done, indicating which were pre-specified. | Methods: Meta-analyses (p. 9) |
| **RESULTS** | | |  |
| Study selection | 17 | Give numbers of studies screened, assessed for eligibility, and included in the review, with reasons for exclusions at each stage, ideally with a flow diagram. | Figure 1 (p.9, uploaded separately) |
| Study characteristics | 18 | For each study, present characteristics for which data were extracted (e.g., study size, PICOS, follow-up period) and provide the citations. | Table 1 (p. 10, p. 22-24) |
| Risk of bias within studies | 19 | Present data on risk of bias of each study and, if available, any outcome level assessment (see item 12). | Results, Table 1, Table 2 (p. 10-12, p. 22-25) |
| Results of individual studies | 20 | For all outcomes considered (benefits or harms), present, for each study: (a) simple summary data for each intervention group (b) effect estimates and confidence intervals, ideally with a forest plot. | Table 1 (p. 22-24) |
| Synthesis of results | 21 | Present results of each meta-analysis done, including confidence intervals and measures of consistency. | Results, Figure 2, Figure 4 (p.10-12, uploaded separately) |
| Risk of bias across studies | 22 | Present results of any assessment of risk of bias across studies (see Item 15). | Results (p. 10-12) |
| Additional analysis | 23 | Give results of additional analyses, if done (e.g., sensitivity or subgroup analyses, meta-regression [see Item 16]). | Results (p. 12) |
| **DISCUSSION** | | |  |
| Summary of evidence | 24 | Summarize the main findings including the strength of evidence for each main outcome; consider their relevance to key groups (e.g., healthcare providers, users, and policy makers). | Discussion (p. 13) |
| Limitations | 25 | Discuss limitations at study and outcome level (e.g., risk of bias), and at review-level (e.g., incomplete retrieval of identified research, reporting bias). | Discussion (p. 16-18) |
| Conclusions | 26 | Provide a general interpretation of the results in the context of other evidence, and implications for future research. | Discussion (p. 18) |
| **FUNDING** | | |  |
| Funding | 27 | Describe sources of funding for the systematic review and other support (e.g., supply of data); role of funders for the systematic review. | Provided in the submission system |

*From:*  Moher D, Liberati A, Tetzlaff J, Altman DG, The PRISMA Group (2009). Preferred Reporting Items for Systematic Reviews and Meta-Analyses: The PRISMA Statement. PLoS Med 6(6): e1000097. doi:10.1371/journal.pmed1000097

For more information, visit: **www.prisma-statement.org**.

Page 2 of 2
